# Retinal Ganglion Cell Replacement in Glaucoma Therapy: A Narrative Review

**DOI:** 10.3390/jcm13237204

**Published:** 2024-11-27

**Authors:** Ewa Kosior-Jarecka, Andrzej Grzybowski

**Affiliations:** 1 Department of Diagnostics and Microsurgery of Glaucoma, Medical University of Lublin, 20-079 Lublin, Poland; 2 Institute for Research in Ophthalmology, Foundation for Ophthalmology Development, 60-836 Poznan, Poland; ae.grzybowski@gmail.com

**Keywords:** glaucoma, retinal ganglion cells, replacement theraphy

## Abstract

Glaucoma is a leading cause of irreversible blindness worldwide. It leads to the progressive degeneration of retinal ganglion cells (RGCs), the axons of which form the optic nerve. Enormous RGC apoptosis causes a lack of transfer of visual information to the brain. The RGC loss typical of the central nervous system is irreversible, and when glaucoma progresses, the total amount of RGCs in the retina enormously diminishes. The successful treatment in glaucoma patients is a direct neuroprotection by decreasing the intraocular pressure, which enables RGC protection but does not revive the lost ones. The intriguing new therapy for advanced glaucoma is the possibility of RGC replacement with new healthy cells. In this review article, the strategies regarding RGC replacement therapy are presented with the latest advances in the technique and the obstacles that it meets.

## 1. Introduction

Glaucoma is one of the leading causes of blindness worldwide, which will affect 111.8 million people by 2040 [1,2]. It is a neurodegenerative disorder characterized by selective, progressive degeneration of the retinal ganglion cells (RGCs) and as a result, also the optic nerve (ON) [3]. The glaucomatous degeneration of the ON results in cupping, a characteristic appearance of the optic nerve head (ONH) observed in the eye fundus and the gradual deterioration in the visual field (VF), which leads to irreversible visual loss. The biological background of glaucoma is not fully understood, and the factors contributing to its progression have not yet been sufficiently characterized [4]. Glaucoma remains undiagnosed for many years until the advanced stages of its prolonged asymptomatic course. Additionally, it takes many years from the disease’s initial stages to the first typical changes in the visual field. There is some risk factors identified for glaucoma with elevated intraocular pressure (IOP) as the only one that is modifiable. It is highly proven that decreasing the IOP slows down the disease progression. However, an elevated IOP may remain increased for many years in patients, not causing glaucomatous damage and, on the other hand, there are patients with a low IOP and advanced glaucoma [5].

**Figure 1:**
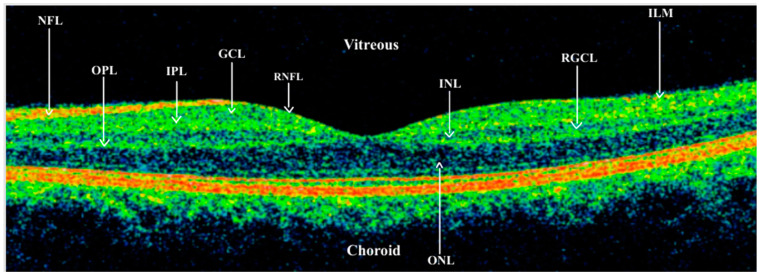
Linear scan obtained using OCT technique from healthy subject in the macular region with marked retinal layers (obtained using Zeiss Cirrus 6000, Carl Zeiss Meditech, Warsaw, Poland). ONL—outer nuclear layer; OPL—outer plexus layer; INL—inner nuclear layer; IPL—inner plexus layer; RGCL—retinal ganglion cell layer; RNFL—retinal nerve fiber layer; ILM—internal limiting membrane.

Although the primary site of glaucomatous injury is not fully clear, the disease leads to a progressive degeneration of the RGCs with their somas and dendrites located in the retina and axons forming the optic nerve [6]. The location of the RGCs in the retina is crucial for understanding the possibilities and difficulties facing replacement therapy. The human retina contains six main neural layers, three of which contain cell somas: the outer nuclear layer (ONL), the inner nuclear layer (INL), and the retinal ganglion cell layer (GCL). The ONL contains the rod and cone photoreceptors; the INL comprises three distinct populations of interneurons: the bipolar cells, the horizontal cells and the amacrine cells; the inner layer of the retina (GCL) is where the RGCs are localized [6]. The distinct retinal layers are well distinguishable in the scans obtained via optical coherent tomography (OCT) [Figure 1].

## 2. Retinal Ganglion Cells

RGCs are larger than most of the other retinal neurons, and their dendrites obtain inputs from all the preceding retinal neurons initializing the visual pathway. Their large-diameter axons transmit the visual signal to the related areas of the brain, located far away from the RGC somas. In the retina, RGC axons converge toward the center, where they form the ON starting at the ONH; the nerve passes the information to the brain centers, mainly the lateral geniculate nuclei in the thalamus, and to a lesser extent to the suprachiasmatic nuclei, the superior colliculi, and other pretectal nuclei. These highly complex and unique connections develop during RGC axon growth, which enables the forming of synapses toward the appropriate target cells [6].

RGCs function as the only output neurons transporting the visual information from the retina to the specific targets in the brain. However, they are also involved in the early processing of visual information. RGCs extract in parallel different attributes of the image: spatial contrast, flicker, color, fine details, motion, absolute light level, and coarse textures [7,8]. Moreover, RGCs are not a homogeneous population; for example, in mice, up to 40 subtypes of RGCs have been differentiated according to the multiple visual functions. The mechanisms underlying this diversity are still not clearly understood [7,9,10]. Each RGC subtype is responsible for the distinct features of the visual transmission to the central brain targets [11]. Moreover, RGC subtypes differ in their capacities to survive and to regenerate after injury [12,13]. The RGC dysfunction or death during glaucoma may cause irreversible blindness because the surviving RGCs are not able to regenerate [13].

During embryogenesis, RGCs are the first cells arising from retinal progenitor cells (RPCs), which are a multipotent cell type that further differentiate into seven cell subtypes forming the neural retina: cone and rod photoreceptors, horizontal, bipolar, and amacrine interneurons, the cell bodies of radial Müller glia and RGCs [14].

### RGCs in Glaucoma Pathogenesis

Since RGCs are thought to be the one of the possible sites of primary injury leading to glaucoma, their repair and replacement have been identified as a crucial target for visual function restoration which could be an effective treatment of the disease. The pathology of glaucoma involves a lot of different cellular processes related to RGCs such as axonal degeneration and the degeneration of dendritic arbors and neuron soma [15]. The degeneration of RGC axons in glaucoma injures the transport of the information to the brain, leading to the loss of the input from the visual pathway in the anterograde direction and the subsequent RGC death, which finally leads to the irreversible loss of vision.

During early glaucoma, a causative harmful factor leads to RGC dysfunction and the activation of the glial cells in the retina and ON. It is believed that at this stage of glaucoma, possible neuroprotective strategies may concentrate on restoring the normal homeostasis between injured RGCs and their environment. As the disease progresses, the subsequent early degenerative events include the remodeling of the cytoskeletal and synaptic structures throughout the RGC projection and increased inflammatory signaling from resident glial cells. At this early stage, possible neuroprotective therapies are thought to repair the damage, which probably remains still reversible at the cellular and molecular level and aim to decrease the level of inflammation to prevent additional damage. Ongoing glaucoma includes the irreversible loss of RGCs and their axons with glial scarring in the ON, which constitutes the obstacle to axonal regeneration. Therefore, at late stages, neuroregenerative and replacement therapies to restore visual function are needed [16].

In some cases, neuroprotective treatment may protect RGCs before the occurrence of apoptosis. The strategy of having a neuroprotective potential has been broadly proven in a plethora of multicenter studies focused on decreasing the IOP. However, RGC regenerative or enhancing strategies with IOP decline or the possible application of direct neurotrophic factors cannot be a viable solution for many patients with advanced glaucoma. In such cases, only cell replacement therapy replenishing the lost RGCs can constitute a treatment option providing vision restoration. However, RGCs are incapable of self-renewal, so the replacement of injured RGCs with healthy cells has always been a proper therapeutic target [17,18].

Therapeutic strategies that support visual restoration have focused on protecting RGCs from degeneration, promoting RGC and axon regeneration after injury, and reestablishing their correct projection relationships [13]. However, every part of this strategy meets obstacles when preserving/restoring RGCs and regenerating their axons are considered using the following approaches: (1) increase the RGC survival or supplement the retina with new RGCs, (2) overcome the inhibitory potential of the optic nerve environment for neurogenesis, (3) enhance/create RGC axon growth, and (4) create the right RGC connections with their typical targets in the brain [19]. Vision restoration at the translational level in clinics has been hindered by several challenges such as the source of the stem cells, differentiation into appropriate cell types, transplantation approaches, survival, and the functional integration of cells into the target retina. Additionally, to establish functional neuronal connectivity, an optimal microenvironment mediated by the trophic factors is crucial. Similarly, the survival of the transplanted cells also depends on a healthy retinal microenvironment [20].

Moreover, the problem with the restoration of vision is that it requires transplanted RGCs to grow normally functioning axons which could extend beyond the ONH, their remyelination by oligodendrocytes, and the proper guidance toward the terminals in the CNS where new synaptic formation should occur. RGCs do not act as an isolated unit; their function relies on cells’ both intrinsic and extrinsic factors changing during glaucoma to determine the cell fate. In fact, RGCs rely on numerous cell populations for survival in the visual pathway. The latest research showed that astrocytes straightaway take action to deliver neuroprotection during stress by using alternative metabolic resources [16].

On the other hand, neuroprotective strategies with the application of transplanted cells which defend the endogenous neural tissue have demonstrated encouraging outcomes in animal models of some retinal disorders (reviewed in [21]). Initial studies transplanting hippocampus-derived neural progenitor cells into the retina showed some structural integration. Grafting these cells into mature rodent eyes after iatrogenic retinal scarring or ischemia-reperfusion insult, evolved donor neurite integration and the cellular expression of microtubule-associated protein 2 (MAP2) and glial fibrillary acidic protein (GFAP) [22]. These findings provide initial evidence for the possibility of the therapeutic application of cell transplantation within the inner retina. Since then, studies have been accomplished evaluating the intravitreal transplantation of different cell types, including embryonic stem cells (ESCs), Müller glial-derived cells, mesenchymal stem cells (MSCs), and primary or stem cell-derived RGCs [23]. The possible neuroprotective pathway of action may involve the secretion of trophic factors and/or the modulation of inflammatory processes. Moreover, cell transplantation may improve the endogenous repair mechanisms via the enhancement of inhibitory signals to encourage axonal regeneration and neuritic growth [24].

Although encouraging, the progression from the initial basic research studies to functional and clinically relevant RGC transplantation requires more steps toward a significant increase in the efficacy of the integration of transplanted neurons within the retina [23]. Below, we review the most important achievements in the dynamically evolving field of RGS transplantation. A lot has been achieved; however, still a lot needs to be addressed to apply these results to restore the vision of blind glaucoma patients. Unfortunately, nowadays, there are neither neuroprotective nor axogenic therapies capable of restoring lost visual pathway connectivity in degenerative retinal disease nor translatable techniques to replace the lost RGCs and photoreceptors [25].

## 3. Source of Cells for Transplantation

The RGC is incapable of self-renewal, so the only possible treatment is the replacement of injured RGCs with healthy cells. One of the crucial questions that endures in cell transplantation is whether to use allogeneic or autologous cell sources for the derivation of cell therapy products.

The first idea that comes to mind is allogeneic RGC transplantation, which has been considered in research isolating RGCs from the retinas of recently deceased persons for transplantation into patients [26]. However, RGC transplantation therapy needs a more sufficient and possibly a more vigorous source of healthy RGCs to become an applicable treatment option [27]. Previous RGC transplantation research has produced diverse outcomes and success (reviewed in [28]), but thus far, unfortunately, has resulted in narrow clinical application. The inconsistency in transplantation efficacy may be the result of the availability and purity of a reliable donor cell source, the diversity in a tested host and graft species, and the mode of cell delivery [28].

In a relatively new branch of studies, allogeneic stem cells can be arranged not to have the Human Leukocyte Antigen (HLA) genes, which make them “cloaked” to the host immunological system. According to this theory, one donor could be the source of blindness-reversing treatments for a lot of patients. These involve stem cells obtained from blastocysts—human embryonic stem cells (hESCs) or induced pluripotent stem cells (iPSCs). Such strategy passes the exam in the case of retinal pigment epithelial (RPE) cells: hESC-derived RPE cells were the first pluripotent stem cell-derived RPE cells transplanted into the eyes of patients [29].

In contrast, autologous cell sources focus on the application of the patient’s own cells. First, the patient’s own retinal cells with the potential for regeneration could be used. Troeppe et al. showed that adults’ retinal stem cells may be found in the pigmented ciliary margin, indicating that they are homologous to the cells found in the ocular germinal zone of other nonmammalian vertebrates [30]. However, there is a lack of evidence showing that they can replace damaged RGCs. In fish and amphibians, similar cells are located in the poorly differentiated ciliary marginal zone and are responsible for providing the recruitment of cells to the growing retina and are involved in the regeneration of the injured retina. In mammals, this region is represented by a limited number of cells localized in the ora serrata [31].

Of the endogenous retinal stem cells, Müller glia were successfully induced to dedifferentiate into RPCs able to transform into multiple retinal phenotypes. Ciliary epithelial-derived stem cells are multipotential and self-renewing retinal RPCs which may be obtained from the pigmented ciliary epithelium of the retina with the potential for differentiation in vitro into photoreceptors. The RPE layer is able to generate the remaining layers to create new retina in some animals and, in humans, contains a tiny group of stem cells that can differentiate into new RPE cells and cells with a neuronal phenotype. Transplantation and manipulation within endogenous retinal stem cells have the capability to cure retinal degeneration; however, their application is probably restricted to RPE and photoreceptor replacement therapies. RGC replacement seems to be much more refractory to such strategies [23].

Studies undertaken on whole eye specimens or on vitreous samples obtained during vitrectomy showed that human non-pigmented ciliary epithelium cells (NPCECs) proliferate within the vitreous base in the proliferative vitreoretinopathy [32]. Too et al. described the spontaneous formation of cells migrating from human equatorial retinal explants obtained during vitrectomy for retinal detachment. These migrating cells, probably derived from human equatorial Müller cells, expressed markers suggesting that they re-entered a cell cycle. Such retinal explants are plentiful in retinal units, which may be a promising source of RPCs [33]. Additionally, some studies have proved that mammalian Muller cells have the ability to reprogram into a proliferative progenitor-like state via the application of diverse intervention methods [34]. Some studies reported that NPCECs can differentiate into ganglion, amacrine, and Muller glia cells [35]. Moreover, NPCECs’ secretome may induce RGC differentiation in vitro, and TGF-beta 1 is one of the neurotrophic factors pivotal in this process [36].

Since RGCs differentiate straight from stem cells, it may offer a promising area for research to introduce RGCs derived from stem cells, which constitutes a new attitude with an ample source of RGCs efficiently available for replacement therapy [20]. Stem cells are defined as undifferentiated cells with the ability of proliferating, self-renewal, and reproducing identical multipotent stem cells indefinitely in their undifferentiated state and being able to produce one or more differentiated cell types [27]. They constitute one of the possible therapeutic strategies to be applied if tissue repair and regeneration is required, and one of such cases is glaucoma [37]. The studies on RGC-ablated mouse models showed that stem cells transplanted into the retina could survive, differentiate into an RGC lineage, and to some extent integrate into the GCL to refine the visual function [20]. In addition, the derivation of RGCs from stem cells is a promising modality in restoring vision when the transplanted stem cells are able to express RGC-specific proteins and to develop some of the RGC morphology features [27].

Two types of stem cells may be found in humans: ESCs found in blastocysts and adult stem cells or pluripotent cells that can be found in a wide variety of adult tissues. The stem cells may also be obtained by inducing mature cells to re-differentiate into the pluripotent status via molecular manipulation and are then called iPSCs. Most iPSCs are manufactured via the application of viruses (lentiviruses and retroviruses) carrying the genes responsible for the transcription factors needed in adult cells for modification. These genes will then enable transcription and translation into a protein initializing the mature cell nucleus to acquire an embryonic state [37].

In vivo, the differentiation of stem cells into RGCs is regulated by several transcription factors such as Brn3, Ath5, and Notch. Brn3 and Ath5 play a pivotal part in the differentiation of RGCs, and their levels increase during eye development [38]. Notch is a negative regulator of RGC differentiation with decreased levels during normal eye development. The addition of Brn3, Ath5, and the Notch antagonist is one of the applied strategies to obtain RGCs from stem cells [14]. Additionally, the studies identified various neurotrophic pathways and the differentiation of stem cells into RGCs consisting of fibroblast growth factor, insulin-like growth factor [39], bone morphogenetic protein, nodal, and Wnt signaling pathways [37,40].

The research on stem cell technologies has made rapid progress, from the isolation and culture methods of ESCs to the remarkable improvement of the methods of the differentiation of pluripotent cells into retinal lineages in vitro [6].

### 3.1. Embryonic Stem Cells

ESCs are capable of indefinite proliferation by following the cycles of the natural development and differentiate into any cell types of all three germ layers (ectoderm, mesoderm, and endoderm). Recent studies have reported the successful production of human RGCs from hESCs [27].

A few groups have attempted to transplant different types of RPCs into rodent eyes with rather poor results because of insufficient survival and limited evidence of functional RGC replacement. In preclinical studies, RPCs obtained from hESCs were able to integrate into the murine GCL and expressed the RGC marker Brn3a, and an increase in ONL thickness was detected [41]. RGCs derived from mice ESCs and transplanted into the eyes of recipient mice, that had performed the previous NMDA-mediated depletion of endogenous RGCs, were able to survive for at least 12 weeks, grow long neurites that laminated within the IP, and formed synaptic structures [42]. HESC-derived RGCs transplanted into rodent eyes have been reported to localize to the GCL. However, these cells were not able to grow neurites and to express RNA binding protein. Recently, a crucial study showed moderate success in transplanting primary mouse RGCs into rodent eyes, describing quite rare instances of mature RGC morphology, visible synaptogenesis, and functional electrophysiologic responses to light despite an insufficient survival rate [23]. In another study on non-human primates, the subretinal transplantation of retinal organoids obtained from hESCs was well tolerated, and the transplanted cells were found to be integrating into the retinal injury site after laser ablation [41].

Despite the vast promises of hESCs’ application, the possible risk related to the formation of tumors and ethical arguments regarding the usage of human embryos are the typical concerns that constrain their application at the moment. Moreover, the development of iPSC technology has caused a reduction in the use of hESCs [27].

### 3.2. Induced Pluripotent Stem Cells

In 2006, Takahashi and Yamanaka [43] published their study in which mouse and adult fibroblasts were successfully reprogrammed into a pluripotent state after the application of specific transcription factors (Oct3/4, Klf4, Sox2, and c-Myc) delivered thanks to transfection mediated by the retroviruses. The cells obtained as the result of this modification, iPSCs, were able to form colonies morphologically similar to ESCs and with the potential for differentiation into three germ layer cell lineages. Since iPSCs could be obtained by reprogramming of the patient’s somatic cells, the individual genomic information could be maintained [43,44]. It was a breakthrough in the field of stem cell research since these patient-derived iPSCs may be used as an ideal in vitro model for the studies on genetic diseases, and thus, they may in future have a favorable role in the work on personalized treatment without the need for using embryos [45]. Potential non-retinal-derived mature stem cell-derived strategies which have evolved as a cure for retinal degeneration include NSCs and MSCs obtained from either bone marrow, dental pulp, or adipose tissues [25]. There have been many advancements in the technology applied for generating and manipulating iPSCs. The derivation of iPSCs from somatic cells has made them an encouraging therapeutic option in regenerative medicine without the risk of immunological rejection [27]. Additionally, the application of viral vectors to enable the introduction of the reprogramming factors was substituted by non-viral methods, such as plasmid, protein, and miRNA as new vectors to deliver the transcription factors without the possible risk of the subsequent mutagenesis of the recipient cells. Other studies showed that the addition of small molecules, such as valproic acid, butyrate, AZA5-aza-cytidine, vitamin C, MEK inhibitor, transforming growth factor-β receptor inhibitor, ROCK inhibitor, and GSK3β inhibitor could increase the reprogramming efficacy and finally replace the application of certain transcription factors in the methods of obtaining iPSCs [44]. Moreover, the number of reprogramming factors was reduced, making the process more effective and cost-minimal [45].

In recent years, iPSCs have been broadly studied for neurological diseases mainly because of the limited possibilities to study the human brain directly. iPSCs can be directly obtained from patients with specific neurological diseases (such as Parkinson’s disease, Alzheimer’s disease (AD) [46]. In glaucoma, recent research has shown that human iPSCs have the potential to differentiate into RGCs. For example, in their studies, Li et al. [47] forced human iPSCs to create a three-dimensional (3D) retina [48]. Afterwards, the RGCs were generated from this human iPSC-derived neural retina. Additionally, Tanaka et al. [49] obtained self-induced RGCs with growing axons from human iPSCs. Moreover, lately, there have been reports showing the successes in the production of human RGCs from human pluripotent stem cells and human Tenon’s capsule fibroblast-derived iPSCs [27].

On the basis of transcriptome profiles, a few iPSC-RGC subtypes have been distinguished. It was possible to obtain the transcriptomes of all iPSC-RGCs from typical iPSCs without the need for additional gene manipulation, but they may be different during the development or maturation of specific cells. These differences in gene expression may be used as potential biomarkers for the classification of RGC subsets. It is possible that understanding the RGC diversity will help to identify the different RGC subtypes with the possibly different susceptibility to degeneration or an injury during the subsequent stages of glaucoma and/or optic neuropathies, and consequently, the identification of RGC subpopulations susceptible or resistant to ON injury may enable the planning of precise and individual treatment strategies [46].

RGCs differentiated from human iPSCs were reported to survive for 5 months post-transplantation, migrate into the endogenous RGC layer, and extend “wild type like” dendritic arbors into the IPL [42]. Johnson et al. [42] performed electrophysiology in five of six iPSC-RGCs and demonstrated the response to full-field photopic light stimuli. However, the RGC response to the light stimulus is driven by photoreceptors or may come from melanopsin-expressing RGCs themselves. The natures and kinetics of both types of responses significantly differ, with the melanopsin-driven one being much slower in origin and recovery. Surprisingly, in the case of hiPSC-RGCs, the exposure to light created a distinct rise in their firing rate, which declined to the basic values in less than a second after the light was extinguished. Such kinetics are not similar to the typical kinetics of melanopsin-driven responses, so hiPSC-RGC responses should be classified as photoreceptor-driven [28]. The efficiency of transplanted RGC integration suggests normal synaptic connectivity to the inner retinal plexus [42].

Stem cell differentiation is a complex and slow process, and as a consequence, the obtaining of RGC-like cells from stem cells constitutes a real barrier when stem cell-based therapies of glaucoma are considered. To demonstrate this problem, lots of different factors need consideration: more than forty diverse RGC subtypes have been described without clear features enabling one to distinguish them from other types of neurons, but also in vitro-obtained RGCs do not have specific signature characteristics helping to distinguish them from other neurons. Moreover, there are neither definitive proteins or RNAs that are entirely expressed in RGCs nor distinct electrophysiological features to discern RGCs from other neurons. Therefore, a lot of scientific discoveries are required to be made before RGC creation becomes a process that can enable the delivery of effective RGC differentiation methods [28].

Additionally, some ethical concerns regarding the application of iPSCs are pointed out. The usage of iPSCs for research and therapeutical reasons could cause inequity in the access to healthcare, as nowadays the technology to produce and apply these cells remains high-priced and not broadly accessible [45]. Moreover, there are discussions about the limitation of genetic diversity, which jeopardizes the fairness and may hinder the pace of biological breakthroughs. There is also the possibility of the iPSC application in controversial sectors, such as the creation of genetically modified organisms or human cloning [45].

The advantages and disadvantages of the different sources of RGCs are summed up in Table 1.

### 3.3. Two-Dimensional (2D) Cell Culture and Three-Dimensional (3D) Organoid

The differentiation and the culture of iPSCs resemble the normal development of the eye [44]. At the beginning, the embryoid bodies (EBs) are established with later OV-like structures or neurosphere formation. EBs are cellular aggregates containing a combination of ectodermal, mesodermal, and endodermal cells, which reflect the three germ layers. OV-like structures or neurospheres are proliferative cellular aggregates of neural progenitor cells differentiating later into neurons or glia. Initially, research showed that the dynamic behavior and critical development of the checkpoints in RGC induction are crucial in the confirmation that the RGCs have established a lineage and followed the typical stages of eye development [44]. The most ubiquitous type of cell culture created during the studies is the 2D culturing system, which involves single-layer cell growth on a flat surface [50]. The application of iPSCs in 2D culture may give insights into the processes connected with neurotransmission within the CNS, and the differentiation of particular cells such as astrocytes, neurons, and microglia [51] subsequently enables a better understanding of the molecular and genetic conditions of neurological diseases [52].

Although the 2D type of cell culturing is broadly used and constitutes an important tool in basic science studies, in fact its role in disease modeling is rather limited since it does not correctly reflect the communications between cells, the dynamic situation of the in vivo environment, and organ- and tissue-level complexity in detail. The reason for this is that 2D cultures have the ability, so far, to differentiate into only one cell type in such mono-layer system [45]. It additionally causes an artificial behavior of these cells, such as atypical proliferation and/or differentiation, and decreased cellular interaction compared to in vivo conditions [53,54]. In spite of these above-mentioned limitations, 2D cell culturing constitutes a useful laboratory method, which has been significantly improved in recent years via the techniques for analyzing and characterizing cells, such as high-content imaging and transcriptomics, which in turn enable more detailed studies on the characteristics of cultured cells [45,55,56].

The conventional 2D cultures have been developed for the attachment and proliferation of iPSCs. On the other hand, the three-dimensional (3D) culture system has become more popular, thanks to its capacity to self-organize. Additionally, it does not need so many extrinsic growth factors, and the intrinsic pattern seems to resemble the normal development of the eye. The used 3D culture techniques involve the suspension of the culture and then cell encapsulation in gels and further cell culturing in the scaffolds [44]. Organoids are 3D structures obtained from stem cells or tissue explants that can self-organize into structures similar to specific organs. They can display the complexity of cellular configurations, structural orientations, and actions resembling normal in vivo tissue, comprising various cell types, and may be used to investigate disease modeling, organ development, and drug screening [45]. The 3D culture of iPSCs allows for self-organization of the earliest eye structure. These structures include optic vesicles and optic cups, which finally become retinal lineages with photoreceptors, RPE cells, and RGCs within the structure. Under such culture conditions, different retinal cell types are generated, and later organize into a typical retinal layout. Unfortunately, the large-scale culturing or enrichment of specific retinal cells (i.e., RGCs) still seems to be limited [57]. Early retinal organoids (8–12 weeks after induction) include all the types of retinal cells and layers, which can be found in a typically developing human fetal retina [58].

As in normal human developing retinas, rod photoreceptors are far more frequent than the RGC-like cells. The average number of such RGC-like cells has been evaluated to be 0.1–30% of the total cell count. Moreover, the number of RGC-like cells tends to decline with time, which is comprehensible knowing that early RGCs undergo two apoptosis cycles in normal development. During this period, RGCs are strictly dependent on brain trophic support, which is not present in organoid cultures [59].

The usage of both ESCs and iPSCs has shown the capacity for differentiation into 3D retinal organoids [60,61], and thus, retinal organoid techniques could establish the main approach in transplantation studies [58], as recent research efforts have successfully shown [62,63]. To delineate the contribution of distinct signaling pathways of RGC differentiation, Dorgau et al. [64] studied the influence of Laminin c3 in RGC differentiation in retinal organoids obtained from hPSCs. Other studies evaluated RGCs obtained from 3D retinal organoids as RGC transplantation media [65]. Summarizing, retinal organoids are now a promising direction in the studies, and hopefully, one day, scientists will find solutions for overcoming the problems with RGC replacement strategies, and result in vision restoration methods [58]. However, comprehensive, effective, and safe protocols for organoid manipulation are waiting to be developed to produce RGCs in large quantities and to be applied in human therapies [27].

The viability and gaining the function via transplanted cells are crucial to the success and effectivity of cell transplantation within the transplanted microenvironment [66]. It has been shown that the application of 2D or 3D tissue-engineered scaffolds constitutes an effective strategy to defeat the limitations of cell transplantation since cell suspension seems to have a smaller immune advantage for substrates provided as a whole structure. Additionally, the total differentiation and correct integrity of the supporting material may be mentioned as further advantages of the scaffold application. Tissue-engineered scaffolds may provide a physical base for cell transportation, viability, and integration [67]. So, the scaffold application as a cell transportation mode shows a promising way to achieve success in cell transplant strategies in the therapy of glaucoma and other retinal degenerative disorders [68]. This is because the scaffolds are able to create the natural microenvironment typical for neural tissues, and as a consequence, help in the restoration of broken axonal connections and the RGC replacement [27,69].

## 4. Application of RGCs to the Donor’s Retina

### 4.1. Scaffold Material

All the cell replacement strategies need the transplanted cells to relocate from the distribution site to their final location within the recipient organism. However, RGC replacement therapy encounters specific obstacles: RGCs delivered to the eye need to penetrate within the retina, connect with bipolar and amacrine cells on one site, and connect via axons with the specific targets in the brain [70]. Success critically depends on the survival of the delivered cells, maintenance of their phenotype, and their integration within the proper tissue [70]. The low cell viability and engraftment rates at the transplant location and problems with the maintaining of the injected cells in a targeted area remain the major challenge. It is pivotal to find a scaffold for RGC delivery that additionally enhances regeneration. Without a structural base in the retina, the delivered cells will miss cell–cell and cell–substrate support, which will promote excessive apoptosis. The surviving cells would not have a matrix to be structurally laid out in and would usually migrate from the correct place to nearby host tissue [71]. So, there are attempts to discover an ideal cell-carrier scaffold for transplanted cells which could enhance the ability of RGC delivery, planting newly created dendrites into the IPL, producing axons toward the ONH, and regenerating axons for places a distance away [68]. Moreover, these biopolymeric scaffolds should enhance the guidance of the RGC radial growth from the ONH to the brain [27,72]. An ideal scaffold material would increase the cell viability but disappear within a specific time without inducing an inflammatory reaction harmful for the graft fates [68]. Unfortunately, the studies show that the outer retina environment is unfavorable for the viability of transplanted RGCs [24,27].

Typically, the grafted cells are distributed to the target location in a buffered saline solution (BSS). Questions remain as to whether the cells obtain sufficient chemical and physical stimuli to promote therapeutic success, and whether the cells stay at the target location for enough time to anchor and become integrated within the host tissue [73]. For ocular applications, the biomaterial substitute of BSS should be injectable but simultaneously be able to transform into a solid material in vivo. Lots of hydrogels have such potential; however, only a few can perform spontaneous covalent cross-linking in situ to enable the right control of the degradation rate, gelation, and mechanical behavior [74].

Many biomaterials may help in sustaining the planned cell phenotype, differentiation, and proliferation [75]. However, another discussive and difficult point in RGC replacement attempts is the diversity between the conditions of the in vitro culture and in vivo systems. The cells in the lab grow in the ideal culture conditions to achieve the highest survival rate; after transplantation, in vivo, the cells encounter challenging degenerative conditions. If RGCs undergo successful encapsulation within the gels, they become provided with a 3D protective microenvironment protecting them from the direct exposure to the hostile host tissue conditions [71].

A lot of biopolymer materials, mostly carrying the functional ester group, have been studied as a scaffold for the monolayers obtained from stem cells [76,77], namely polylactic acid (PLA) [78], polylactic-co-glycolic acid (PLGA) [79], polycaprolactone (PCL) [80,81], and polyglycerol sebacate (PGS) [82]. The advantage of the mentioned biopolymers is that they keep differentiated cells able to achieve the management of RGCs’ function and structure. The research is focused on scaffold modification to obtain optimal effective RGC growth towards the ONH. Kador et al. [78] obtained a PLA electrospun scaffold imitating the mode of radial RNFL axon paths. PLA-derived scaffolds help to increase the RGC axon growth, improving the regeneration of long-distance connections to the CNS. Lately, Li et al. [83] obtained PLGA-based scaffolds as a base for human RGCs and described that the RGCs had integrated with the scaffolds and exhibited their structural character. This engineered scaffold was able to mimic the RNFL bundles and to present dendritic arbors, grow axons and a neurite plexus, and exhibit electrophysiological features [27].

In addition to the need for the further advances in the world of scaffolds, surface modification techniques are also crucial for the improvement of the clinical success in RGC replacement therapies. The control of cell differentiation is needed to achieve not only anatomical but also functional regeneration. An appropriate biopolymer scaffold should encourage the correct cell differentiation of the RGCs transported to the retina; however, this is feasible only at the surface area of the scaffold at the nodal points of cellular attachment. Specific surface modifications to biomaterial scaffolds were helpful for the viability of delivered cells by imitating their natural microenvironment and, as a result, improving the biocompatibility and enhancement of phenotype expression in comparison to unmodified scaffolds [27,84].

### 4.2. Intraocular Barriers

The intraocular transplantation of the prepared replacement RGCs for retinal therapy can be obtained via two modes of application, either subretinally or intravitreally, with each approach having its pros and cons. First, subretinal injections put cells nearby the outer layers of the retina and near to abundant blood circulation, which is desired in the case of photoreceptor therapies. On the other hand, intravitreal injections have an easier technique, familiar to every vitreoretinal surgeon, and enable the straight access to the inner retinal layers. Most studies into intraocular grafts are related to subretinal approaches, partially because of being focused on the degenerative diseases of photoreceptors [42]. However, in the case of inner retinal disease with glaucoma as an example, intravitreal injections constitute probably the more applicable approach than subretinal injections. Unfortunately, the studies regarding subretinal application described both cellular factors and ECM molecules as inhibitory to graft migration. However, it remains elusive whether they have the same role after intravitreal placement of the graft [42].

The subretinal injection typically used in photoreceptor transplantation seems to be less effective in RGC replacement strategies because the transplanted RGCs need to pass the whole retina with its many layers to target the RGCL and then grow axons toward the RNFL. On the contrary, intravitreal transplantation enables a straighter approach to the internal layers of the retina. However, it causes new problems with the distribution of the injected cells into a large three-dimensional vitreous cavity and behind physical barriers not existing when using the subretinal way of cell administration [85]. Additionally, for the transplantation of RGCs into the eye, intravitreal injection is the most favored way of administration as it does not disrupt the blood–retinal barrier, and it avoids systemic exposure. However, in the large vitreous space, the control of the distribution of the injected RGCs to a targeted retinal layer remains laborious, and the donor cells may sometimes be found at the posterior lens capsule and ciliary body [28]. It was reported that only about 1% of RGCs administered into the vitreous cavity usually find the targeted retina, and unfortunately, most of them remain outside of the neural tissue [42].

Prior studies have reported that the ILM constitutes the main barrier inhibiting the integration of transplanted RGCs to the retina. The ILM is a basement membrane mostly comprising laminin, collagen IV, and other ECM proteins [70]. Other studies have shown that rather more reactive gliosis than the ILM may result in the failure of the RGCs’ implantation via the vitreous approach [86].

Lately, the ILM has attracted more clinical interest with an increase in the recognition of its role as a barrier which may impede the breakthroughs with novel transplanting ophthalmic strategies. The ILM may hamper retinal targeting due to intravitreally delivered gene therapy vectors but also the retinal integration of cell transplants. The ILM also constitutes a barrier to intravitreally injected nanoparticles and antibodies [23]. Intravitreally transplanted cells are blocked at the level of the ILM surface without approaching the host retina.

Zhang et al. provided the straight proof that recognizes the ILM as a crucial barrier hampering transplanted human RGCs from integration [86]. Using organotypic mouse retinal explants with transplanted hES-RGCs, they found that hES-RGCs encountering an unbroken ILM were not able to grow neurites toward the recipient retinal parenchyma. On the contrary, transplanted RGCs integrated neurites into the IPL in areas where the ILM was mechanically broken. Localization of the RGC dendrites to the IPL is a prerequisite for the further development of working synaptogenesis with amacrine and bipolar cells. An intact ILM seems to create a pivotal barrier to hES-RGC neurite ingrowth in the retina, as the mechanical disruption or enzymatic degradation of ILM proteins caused the visible improvement of the retinal neurite ingrowth. So, it seems that novel methods enabling grafted RGC neurites to cross the ILM is basic to the improvement of RGC replacement success [87].

The ILM’s thickness increases with aging and in the course of some diseases such as diabetes as an example [88], which may cause a greater problem with engraftment in patients with age-related optic neuropathies such as glaucoma. The administration of enzymes degrading the ILM may not be obligatory in human patients since the surgical technique of the ILM peeling is a broadly used and safe maneuver in vitreoretinal surgery. On the other hand, the intravitreal application of proteolytic enzymes may be toxic to the adjacent tissue when applied at the concentrations necessary for the clinical digestion of the ILM [85].

The studies on retinal development show that the interactions of neurons with the components of the ECM is obligatory for their growth, maturation, and polarization. Therefore, although the enzymatic disruption or mechanical removal of the ILM improves RGCs’ migration and further neurite implantation into the retina, the ECM molecular pathways seem to be necessary to achieve the real functional integration requiring donor cell polarization, dendrite stratification, and axon pathfinding. Such approach shows that it may be more important to manipulate cell–ILM interactions, rather than to anatomically eliminate the ILM.

Neurons communicate with the ILM through a group of cell surface receptors. The integrin class is the major family of RGC superficiality that is operated by ECM laminins. The removal of integrins or their downstream Cas adaptor proteins in RGCs may effectively result in RGCL disturbance with RGC ectopic migration through the ILM [89]. Therefore, genetic modification in the integrin pathways in donor RGCs may enhance their migration through the ILM and improve their integration after transplantation [85].

## 5. Paracrine Factors

When planning an optimal cell replacement approach, the unique microenvironment existing within the glaucomatous host retinas needs consideration. Retinas changed during glaucoma may on the one hand have a better ability to accept donor hiPSC-RGCs compared to healthy mature retinas [28]. On the other hand, and theoretically more probably, the paracrine environment in the donor retina may be full of potentially toxic substances which may harm the newly transplanted RGCs.

On the other hand, the paracrine effects of stem cells may offer a new all-encompassing approach to the treatment of neurodegenerative diseases [90]. The paracrine effects of MSCs comprise the production of trophic factors, cytokine neurotrophins, and signaling molecules, which may be helpful for the restoration process by promoting angiogenesis and tissue regeneration, but also via the inhibition of apoptosis and fibrosis. They also change the immune response and the inflammatory process, and the major hypothesis explaining the neuroprotective effects of hMSC focuses on their involvement in the modulation of neuroinflammation, a key process initializing RGC regeneration [91].

Human bone-marrow MSCs are currently authorized for allogeneic and autologous application in humans, and their usage has been previously studied in some clinical trials in traumatology, cardiology, and ophthalmology (ocular surface). However, MSCs have not yet been approved for intravitreal injections. Some initial studies showed that the intravitreal application of MSCs is well tolerated and safe in rabbits, and the cells are bioavailable in the vitreous space [90]. The intravitreal application of MSCs in mice models of different ocular disorders, e.g., retinal degeneration, retinal ischemia, retinitis pigmentosa, and also glaucoma, show that the agents produced by the MSCs have beneficial neuroprotective effects. Bone MSCs constitute an optimal source of cells for the therapy of retinal degenerative disorders and can be obtained in response to tissue injury and repair to strive for paracrine neurotrophic influence [92].

Additionally, some factors which could be involved in the modification of the paracrine environment during RGC replacement therapy are discussed. For example, the α-crystallin proteins necessary for maintaining lens transparency are additionally produced in the case of a light-induced injury to the retina, but also during the wound healing process after a retinal tear. Their expression also correlates with an increased RGC survival following optic nerve axotomy [92]. Nath et al. showed that supplementation with recombinant αA-crystallin protein promotes the endurance of primary RGCs after metabolic injury [93]. The other paracrine factor with a suggested neurotrophic and neuroprotective role is VEGF. The studies reported that VEGF is produced by RGCs to enhance their own vitality [94]. Additionally, Müller glia have been reported to produce different trophic agents with the capacity to influence the various stages of the formation of retinal neuronal connection during the differentiation, synaptogenesis, and neuroprotection of photoreceptors and RGCs in the retina [93].

The molecular pathways beneficial for RGC survival and axon regeneration have been studied via the evaluation of the behavior of endogenous RGCs evolving during experimental stress [95]. For example, intrinsic mTOR and CNTF/JAK/STAT signaling improves the RGC endurance and regeneration of axons [96,97]. Insulin signaling [98], the overexpression of Lin28 [99], the production of thrombospondin-1 [100], the deletion of PTEN and SOCS3 [101,102,103], and the blockage of KLF [104] in RGCs may promote axonal regeneration in different models of traumatic optic neuropathy. On the other hand, over 40 negative intrinsic factors influencing RGC regeneration have been found up today [105]. RGC-extrinsic signaling molecules with oncomodulin may additionally improve the axon regeneration in glaucoma models [85,106].

Finally, donor RGCs may be genetically modified to produce neuroprotective factors and change the recipient microenvironment to improve the endogenous cell vitality and decrease ongoing functional and structural injury. Moreover, the intrinsic molecular signaling in donor cells could be changed to improve their own protective mechanisms to resist the harmful conditions in the host [92].

## 6. RGC Axons

RGC replacement provides a possible therapy to restore the vision loss due to glaucoma. The promising results of photoreceptor transplantation studies initially proved that vision restoration may be achievable via mammalian retinal cell replacement [23]. However, RGC transplantation causes many more possible troubles compared to photoreceptor transplantation, as the transplanted cells need to connect via dendrites with bipolar or amacrine cells and grow their axons through the retina to the ONH and finally meet their postsynaptic targets in the CNS [68]. New RGC axons must penetrate deep into the brain to reach the visual cortex of the cerebrum passing the thalamus centers.

Relying on the type and presence of regenerative stimuli, some axons are able to grow sufficiently toward their targets. However, in many cases, the axons do not elongate but take way back toward the soma. Since functional regeneration needs connections between axons and their related targets, there is a need to define the cellular and molecular factors regulating this process in adulthood [107]. At the time of the primary creation formation of visual projections, RGC axons grow fast in vitro or in vivo, and RGCs can regenerate devastated axons in vivo, but mainly for small distances. The axonal ability for regeneration and growth is absent from the early postnatal period at the moment when RGCs enhance dendritic growth and synaptic inputs expand, which is triggered by the contacts between RGCs and amacrine cells [108].

On the other hand, some studies have described that during regeneration, axons follow astrocytes [107]. In the ON, the astrocytes consist predominantly of the fibrous type, which can be further divided into three subtypes according to their morphology: the transverse subtype, with processes projecting perpendicular to the ON; the longitudinal subtype, with processes projecting parallel to the ON; and the random subtype, with processes projecting in both directions [107]. The studies show that during regeneration, RGC axons just follow their closest astrocyte type. Then, these axons travel stochastically in all different directions, in parallel or perpendicular to the ON. In regenerative therapies with the application of CNTF, many RGC axons travel in diverse directions. Other studies have described that some RGCs can extend toward the CNS more efficiently with the microenvironment enriched with different types of regenerative paracrine agents [108].

Despite the common proofs that RGCs can regenerate axons through a peripheral nerve environment, the regeneration of the mature ON is not thought to be possible because of the strong growth-inhibiting influence of CNS myelin. Berry et al. [109] claimed a diverse point of view regarding the peripheral nerve grafting experiments and proposed that Schwann cell-derived trophic agents may be the primary cause. His team studied if grafting an autologous part of a peripheral nerve into the posterior part of the eye bulb could enhance mature rodent RGCs to grow axons through the ON. In fact, the implants caused visible axon growth, showing for the first time that the ON was not a definitive barrier to regeneration in the case of adequate RGC stimulation [108]. Recent studies have reported that a combination of genetic stimulation and modification of the signaling pathway enhances the regenerative processes in the ON before RGCs find their targets in the CNS. The additive application of ephrin molecules, cell-adhesion molecules, proteoglycans, and semaphorin can help the RGC axons to reach the optic chiasm [110]. Moreover, the application of cadherin, ephrin, and the Wnt signaling pathway molecules can stimulate synapse formation and guide them toward brain centers [107].

Unfortunately, the delivery of NTF has no proven efficacy in enhancing the long-term neuroprotection, which is probably caused by the need for regular administration, and additionally, it applies in combination which may cause the receptor down-regulation after therapy. The application of cells releasing NTF naturally or those which have been modified to release glial cell line-derived NTF and CNFT does not improve the situation. On the contrary, the resolution of this down-regulation problem may be achieved via the application of a virus to enhance the expression of BDNF and its receptor TrkB in RGCs which have been proved to have a neuroprotective action in the case of laser-induced glaucoma [111].

Extracellular vesicles (EVs) are membranous microvesicles containing proteins, RNA, or a whole organelle in specific cases [29]. They are secreted by the cells to play important extra- and intercellular roles in many processes such as immune modulation, cellular communication, physiological regulation, and biomolecular transport [112]. Exosomes are a EV subclass related to disease induction and regeneration. The protective effect of intravitreal injection of MSC-derived exosomes in an experimental model of ON injury was reported to have better RGC vitality and axonal regeneration [113,114]. Additionally, Mead and Tomarev have analyzed the potential of MSC-derived agents, cells, and engineered MSCs to improve the injured retina. EVs derived from human BM-MSCs significantly protected RGCs and diminished RNFL thinning in two rodent models of glaucoma via the EV secreted by BM-MSCs and the miRNAs contained within [115]. The delivery of miRNAs via Schwann cell-derived exosomes into neuronal cultures enhanced neuritogenesis, and the possible miRNAs involved were miR-21, miR18a miR-222, and miR182. The capacity of miRNA to decrease the level and action of a plethora of mRNA makes them a probable candidate for RGC neuroprotective therapy, especially in that they can be easily applied to the vitreous cavity to achieve a rapid and focused influence on RGCs [111].

Though a lot of information was obtained from diverse studies of the endogenous RGC axon regeneration processes regarding molecular pathways engaged in the reestablishment of efferent connections [110], the factors inhibiting donor RGCs from dendrite growth toward the IPL and synapsing with amacrine and bipolar cells still remain unclear. The ILM is thought to be a possible candidate for being such an obstacle.

hES-RGC neurites after reaching the retinal microenvironment do not uniquely grow toward the IPL. The specific agents that drive the RGC dendrite pattern of laminar growth during the normal development to and within the IPL involve molecular cues and activity-dependent refinement [116]. Spontaneous electrophysiological activity in organotypic retinal explants is modest, additionally diminishing gradually, so it may have only modest, if any, potential IPL-guided dendrite localization reinforced by neuronal activity [117]. During development, RGC dendrites target pre-patterned IPL afferents [118]. Sublamination within the IPL is driven by the presence of the specific expression of cell surface receptors and their binding to localized lamina-specific ligands, including integrins, plexins, and cadherins which play a pivotal part in correct dendritic guidance and outgrowth [119]. It is highly probably that the control of specific surface receptor expression may help to navigate hES-RGC dendrites toward their correct targets where afferent synaptogenesis would hopefully happen. It remains elusive whether ligand expression remains present within the mature IPL, but the identification of typical dendritic stratification by transplanted RGCs shows that at least a part of the crucial signaling pathway is visible [23].

## 7. Summary

The in vivo transplantation of stem cell-derived RGCs or exogenous primary RGCs is a highly needed strategy in clinics, providing a chance for the improvement of visual acuity for patients with advanced glaucoma. Unfortunately, basic research and clinical trials remain a field in its infancy. However, the early transplantation studies showed light-evoked electrophysiological responses from donor RGCs [32] and an increase in the visually dependent behavior in studied animal subjects, indicating the principal direction for the clinical potential of RGC transplantation. There are several studies regarding RGC axon regeneration and efferent connectivity after injury; however, there is only scant progress in the specific methods to improve the donor cell vitality, guide RGCs toward the targeted retinal layers, or achieve the afferent synaptogenesis of grafted RGCs [85]. On the other hand, there are a plethora of glaucoma patients for whom RGC replacement therapy is the only way to restore their vision. The possibility of renewing the ability to see for our blind patients makes the RGC replacement therapy a matter of great importance.

There are a few challenges to be addressed in the transplantation of stem cells in the therapy of glaucoma before clinical implementation. First is the source of cells: stem cell application causes ethical concerns and potential tumorigenic worries. The application of iPS, on the other hand, does not solve the potential genetic involvement in glaucoma pathogenesis. Additionally, the introduction of the cells into the host retina needs improvement, not only the process of transplantation but also the integration into the proper layer and reaching the brain target. However, despite the many challenges, there is no doubt that stem cell therapy constitutes the most promising approach for advanced glaucoma patients.

## Figures and Tables

**Table 1:** The advantages and disadvantages of the different sources of RGCSs.

Source of RGC	Advantages	Disadvantages
Emryogenic stem cells	Extensive proliferative and differentiation potential Follow the natural process of differentiation	Possible tumor formation risk Ethical considerations (using human embryos) Long-life immunomodulation
Induced pluripotent cells	Pluripotent differentiation ability Possible cell modifications No immunomodulation needed after transplantation	Ethical considerations (human cloning and genetic modification)
